# Editorial: Emerging Challenges of Cardiovascular and Metabolic Dysfunctions in Cardio-Oncology: From Bench to Bedside

**DOI:** 10.3389/fcvm.2020.00148

**Published:** 2020-08-26

**Authors:** Canan G. Nebigil, Michael W. Y. Chan, Tienush Rassaf

**Affiliations:** ^1^Regenerative Nanomedicine, UMR 1260, INSERM, University of Strasbourg, Strasbourg, France; ^2^Department of Biomedical Sciences, National Chung Cheng University, Chiayi City, Taiwan; ^3^Department of Cardiology and Vascular Medicine, West German Heart- and Vascular Center, University Hospital Essen, Essen, Germany

**Keywords:** cardiooncology, cardiotoxicity, antracyclines, biomarkers, GPCR (G protein coupled receptors), epigenetics (methylation/demethylation), HPSC-cardiomyocytes, HDLP

## Introduction

The number of cancer survivors is increasing and up to 30 new cancer therapies are approved each year with only incompletely characterized side effects (1). Many anti-cancer drugs including traditional, new targeted kinase inhibitors and immunotherapies are associated with cardiovascular and metabolic adverse effects and may have dramatic impact on morbidity and mortality (2). The exact mechanisms how chemotherapeutics induce metabolic disturbances are mostly unclear. Chemotherapeutics-induced oxidative stress and mitochondrial dysfunction may promote metabolic disturbance (3).

Cardio-oncology is a relatively new discipline, aiming at finding an optimal balance between the efficacy of anticancer treatments and the management of their adverse cardiovascular and metabolic effects. It includes the prevention, diagnosis, and treatment of these complications in cancer patients. Identifying markers/predictors of disease risk, ensuring safety of novel cancer therapeutics, developing cardioprotective drugs are the emerging challenges in cardio-oncology (4).

Cardiovascular disease and cancer not only share common genetic, cellular, and signaling mechanisms such as chronic inflammation but also exhibit common risk factors such as obesity and diabetes (5). Dyslipidemia, hypertriglyceridemia, altered levels of low-density lipoprotein cholesterol (LDL-C), and low high-density lipoprotein cholesterol (HDL-C) have been observed in cancer survivals (6). In this regard, about half of the cancer survivors have obesity issue (7). Hypertension, Insulin resistance, hyperinsulinemia, and impaired glucose control that directly affect insulin sensitivity have been observed in cancer survivals (8). Chemotherapies that adversely affect metabolism may amplify cardiac and vascular toxicity, and patient management represents a major economical and clinical burden.

In this edition, we covered the expert reviews on biomarkers and signaling pathways of cardiovascular toxicity and metabolic alteration, and characterization of possible target molecules to prevent or treat cardiovascular damages induced by the cancer therapy.

Kumari et al., focused on epigenetic modifications by doxorubicin (DOX) that can either be used as molecular markers for cancer prognosis or represent molecular targets to attenuate DOX-induced cardiotoxicity in cancer patients.

Kluck et al., outlined emerging preclinical evidence that high density lipoprotein and its precursor protein apolipoprotein A1 may also protect against doxorubicin-induced cardiotoxicity.

Schwach et al., described that human pluripotent stem cell derived cardiomyocytes can be used as a screening platform to test cardioprotective agents against anti-cancer mediated oxidative stress generation and mitochondrial dysfunction, disruption of calcium homeostasis, and changes in transcriptome and proteome, triggering apoptotic cell death.

Mrotzek et al., underlined new studies on the mechanisms and severity of radiation-induced cardiovascular side effects and clinical management and treatment options.

Cardinale et al., discussed in particular troponins as a biomarker of subclinical cardiotoxicity and angiotensin-converting enzyme inhibitors (mainly enalapril) to prevent LVEF reduction in case of early detection of cardiotoxicity and prompt heart failure treatment.

Parichatikanond et al., discussed the molecular mechanisms of TGF-β in the pathogenesis of cardiac fibrosis and cancer and provide *in vitro* and *in vivo* evidences regarding antifibrotic and anticancer actions of TGF-β inhibitors.

Schlaak et al. outlined how inherited genetic variants promote differences in mitochondrial gene expression that may contribute to susceptibility of cancer patients to mediated cardiotoxicity.

Lee's et al. group recommended combined managements with control of comorbidities (such as hypertension, hypercholesterolemia, and diabetes, smoking cessation), and close monitoring and discussed use of statins and angiotensin-converting enzyme inhibitors (ACEIs) in the treatment of cardiovascular disorders induced by anti-cancer drugs.

Livingston et al., presented the evidence that understanding of mitochondria-dependent mechanisms of radiation-induced heart dysfunction can help to develop potential therapeutic targets to assist in prevention and treatment of radiation-induced heart damage.

Audebrand et al. emphasized newly identified cardioprotective agents targeting G protein coupled receptors (GPCRs) of adrenalin, adenosine, melatonin, ghrelin, galanin, apelin, prokineticin, and cannabidiol, provoking further drug development studies for the treatment of human heart failure induced by anticancer drugs.

## Conclusion and Future Challenges

The adverse effects of anticancer treatments including cardiovascular toxicity and metabolic syndrome and their relations with the genetic and environmental factors are still needs to be discovered. Research in cardio-oncology should aim at elucidating the mechanisms involved in cardiovascular toxicity as well as metabolic disturbances. A better understanding of the mechanisms of these adverse effects of anti-cancer therapies may lead to the identification of novel targets for drug development. Currently, evaluation of anticancer therapy-induced cardiovascular toxicity and metabolic disturbance have limitations. Therefore, identification of new early biomarkers of subclinical cardiovascular dysfunctions and metabolic disorders is a key challenge. Research in cardio-oncology should also aim at elucidating the efficacity and toxicity of the new cancer treatments. Thus, the coordinated efforts of oncologists, endocrinologists, and cardiologist are required to overcome these life-threatening problems especially in cancer survivals.

We believe that the topic of “Emerging challenges of cardiovascular and metabolic dysfunctions in cardio-oncology” provides new challenges and potential future directions to the readers from basic scientists, cardiologist, endocrinologists, and oncologist in developing field of cardio-oncology.

## Author Contributions

CN wrote the editorial information. MC and TR have been corrected and edited. All authors contributed to the article and approved the submitted version.

## Conflict of Interest

The authors declare that the research was conducted in the absence of any commercial or financial relationships that could be construed as a potential conflict of interest.
